# Changes in click characteristics during ABR recording

**DOI:** 10.1016/S1808-8694(15)31115-0

**Published:** 2015-10-20

**Authors:** Mariana Lopes Fávero, Fernando L. Carvalho Silva, Alfredo Tabith Junior, Fernanda S Nicastro, Monica C. Gudmond, Mauro Spinelli

**Affiliations:** 1 Doctorate in Otorhinolaryngology at FMUSP, physician, otorhinolaryngologist at DERDIC/PUCSP and HSPM, Division of Education and Rehabilitation of Communication Disorders of the Sao Paulo Catholic University (DERDIC/PUCSP); 2 Physician, otorhinolaryngologist, Professor of the PUCSP Speech Therapy College, Medical doctor at DERDIC/PUCSP; 3 Physician, otorhinolaryngologist, Master's degree in Audiology at PUCSP, of the PUCSP Speech Therapy College, General Director of DERDIC/PUCSP; 4 Master's degree on Communication Disorders at PUCSP. Speech therapist at DERDIC/PUCSP; 5 Speech therapy specialist trained at the Santa Casa de Misericordia in Sao Paulo, Speech therapist at DERDIC/PUCSP; 6 Full professor of Phoniatrics at the PUCSP Speech Therapy College, Physician at DERDIC/PUCSP

**Keywords:** topographic diagnosis, auditory neuropathy, hearing loss

## Abstract

**Summary:**

Manipulation of auditory stimuli affect the ABR evoked potentials and aid the diagnosis, particularly in auditory neuropathy patients. Some patients with auditory neuropathy lose evoked otoacoustic emissions over time; in these cases, comparing responses to rarefaction and condensation clicks, and decreasing the stimulus rate can show an extended cochlear microphonism or yield an improved electric potential record.

**Aim:**

To analyze the effect of these click manipulations on the records of potentials of patients with hearing loss as a form of improving the diagnosis.

**Study design:**

A clinical prospective study.

**Patients and Method:**

59 patients with hearing loss underwent ABR recording using rarefaction and condensation clicks at a stimulus rate of 27.7/sec, and rarefaction clicks at a stimulus rate of 3.3/sec. The records were compared to the otoacoustic evoked emission.

**Results:**

Eight (13.53%) patients showed changes in the recorded ABR potentials as a result of manipulating the characteristics of clicks, such as extended cochlear microphonism or an improved record of electric potentials. Five patients had no otoacoustic evoked emissions.

**Conclusion:**

Manipulation of click stimuli can improve the topographic diagnosis of hearing loss, particularly in the group of auditory neuropathy patients with no otoacoustic evoked emissions, where usually, the diagnosis is only possible through the method described above.

## INTRODUCTION

ABR recording or auditory and brainstem audiometry is defined as a set of electric responses generated in a variety of anatomical sites as a result of an external auditory stimulus of any kind. Since Jewett et al. discovered them in 1970, these potentials - together with otoacoustic emissions - have supported the topographic diagnosis of a number of auditory alterations.^1^

However, in certain patients, among them very young children that do not respond or respond inconsistently to behavioral tests, and present no response to evoked otoacoustic emissions and ABR recordings, the diagnosis of cochlear hearing loss is not always aligned with the progression and improvement following treatment, suggesting that other anatomical sites might be involved besides the cochlea.

Clicks are the most frequently used auditory stimuli to produce electrical responses and ABRs. Recently other stimuli have been introduced to improve the diagnosis, such as the tone burst, used to analyze auditory thresholds at lower frequencies not reached by the click. The stimuli facilitate the diagnosis of sloping loss and the adaptation of sound amplification devices in young children. The bone conduction click is particularly important for the diagnosis of hearing loss associated with malformation of the auricle and the middle ear.^2^,^3^

Changes in click characteristics may also add sensitivity to the diagnosis, adding extra information about the workings of the auditory system. Among these changes, two are often cited in literature: 1. polarity inversion of the stimulus, which facilitates visualization of cochlear microphonism (electric potential generated mainly by external ciliated cells); 2. changes in the click presentation frequency, which is important for analysis of neural synchronism.^4^,^5^

Rarefaction clicks initiate cochlear potentials with different polarities and neural/brain stem potentials with lower latency and slightly higher amplitude, compared with those initiated by condensation clicks, while higher stimulus presentation frequencies reduce wave reproduction and clearness, particularly those generated in distal sites. None of these changes significantly affects the interpretation of results in individuals with no hearing loss.1 However, in subjects with hearing loss and especially individuals with auditory neuropathy (AN), where changes are found between the internal ciliated cells and the auditory nerve, these changes can markedly help the diagnosis.^6^

The aim of this study was to assess the effect of these two click parameter changes in patients with hearing loss, as a form of improving the topographic diagnosis.

## SERIES AND METHOD

The project was approved by the Research Ethics Committee of Derdic and of the Sao Paulo Catholic University.

We conducted a prospective clinical trial involving 59 hearing loss patients (mean age 11.3 years, standard deviation ± 8.5 years) that sought the medical unit of the DERDIC/PUCSP between August 2002 and February 2004.

The inclusion criterion was the presence of severe hearing loss demonstrated by auditory tests done prior to the first consultation in our institution.

Participants underwent a medical consultation, an audiometric exam (with the appropriate technique for each age group), the immitance test, recording of transient otoacoustic emissions HPT (TOAE) and distortion product (OAEDP), and ABR.

Clicks were presented through insertion phones with the following characteristics to record ABR results:

1. Rarefaction clicks, presentation frequency of 27.7 stimuli/sec (standard)
2. Condensation clicks, presentation frequency of 27.7 stimuli/sec
3. Rarefaction clicks, presentation frequency of 3.3 stimuli/sec

Reference electrodes were placed over the right mastoid (A2) and the left mastoid (A1); the active (Fz) and the ground (Fpz) electrodes were placed over the forehead. The test recorded potentials from the ipsilateral mastoid to the stimulated ear during 12 msec after the beginning of each click until an average of 1024 accepted. Response reproducibility was observed for each parameter and for each intensity level until reaching the patient's electrophysiological threshold. The auditory stimulus conduction tube was occluded to differentiate between electrical artifacts and auditory responses for any image suggesting cochlear microphonism.

Data was compared within each test and with TOAE and OAEDP responses.

## RESULTS

Of 59 patients with severe or deep hearing loss included in this study, 8 (13.56%) showed changes in ABR recordings compared to tracings obtained with the standard click when the stimulus polarity was inverted and/or when the presentation frequency was changed. Three of these patients presented TOAE and OAEDP. The remaining 51 patients (84.44%) presented electrical potentials within audiometric thresholds, regardless of the type of click, and no TOAE and OAEDP.

Table 1 shows the changes on tracings with the standard click in those 8 patients, and compares them with TOAE and OAEDP results. Case 4, right ear, Case 4, left ear, Case 6, right ear, Case 6, left ear, Case 7, left and right ears illustrate some of the findings obtained with changes in click parameters.

**Table 1:** Results of OAEs and ABRs with different clicks.

Patient/age	OAET/DP	Click: 27.7 Rarefaction	Click: 27.7 Condensation	Click: 3.3 Rarefaction
1)WM 4.5 years	Absent	Absent	CM present RE up to 4mseg LE up to 2,5msec	No change
2)MP 3.6 years	Absent	Absent	CM present RE up to 4,5msec LE up to 2msec	Wave V bilaterally
3)MLR 19 years	Present	Absent	CM present RE up to 3,5msec LE up to 4msec	No change
4) JMP 3.5 years	Absent	Absent	CM present RE up to 3msec LE up to 2msec	Wave V bilaterally
5) GMS 3.2 years	Absent	Absent	CM present RE up to 2,5msec LE up to 2msec	No change
6) CARB 6.5 years	Absent	Absent	CM absent	Wave I and II in the RE and wave I in the LE
7) ABS 3.2 years	Present	Absent	CM present RE up to 2msec LE up to 4msec	No change
8) SSS 2.8 years	Present	Absent	CM present RE up to 1.5msec LE up to 1.5msec	No change

**Case 4, right ear:**
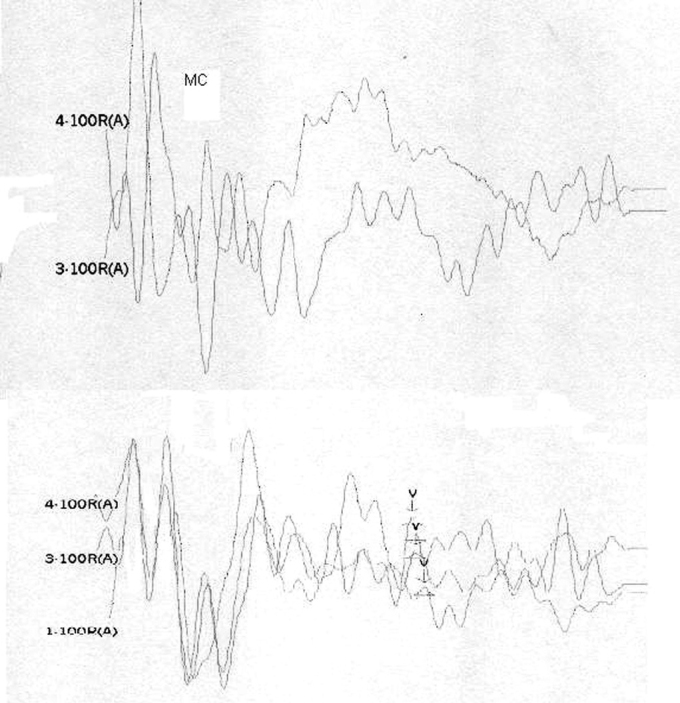
Following stimulus polarity inversion, cochlear microphonism (CM) appears; with a reduced stimulus frequency, wave V appears.

**Case 4, left ear:**
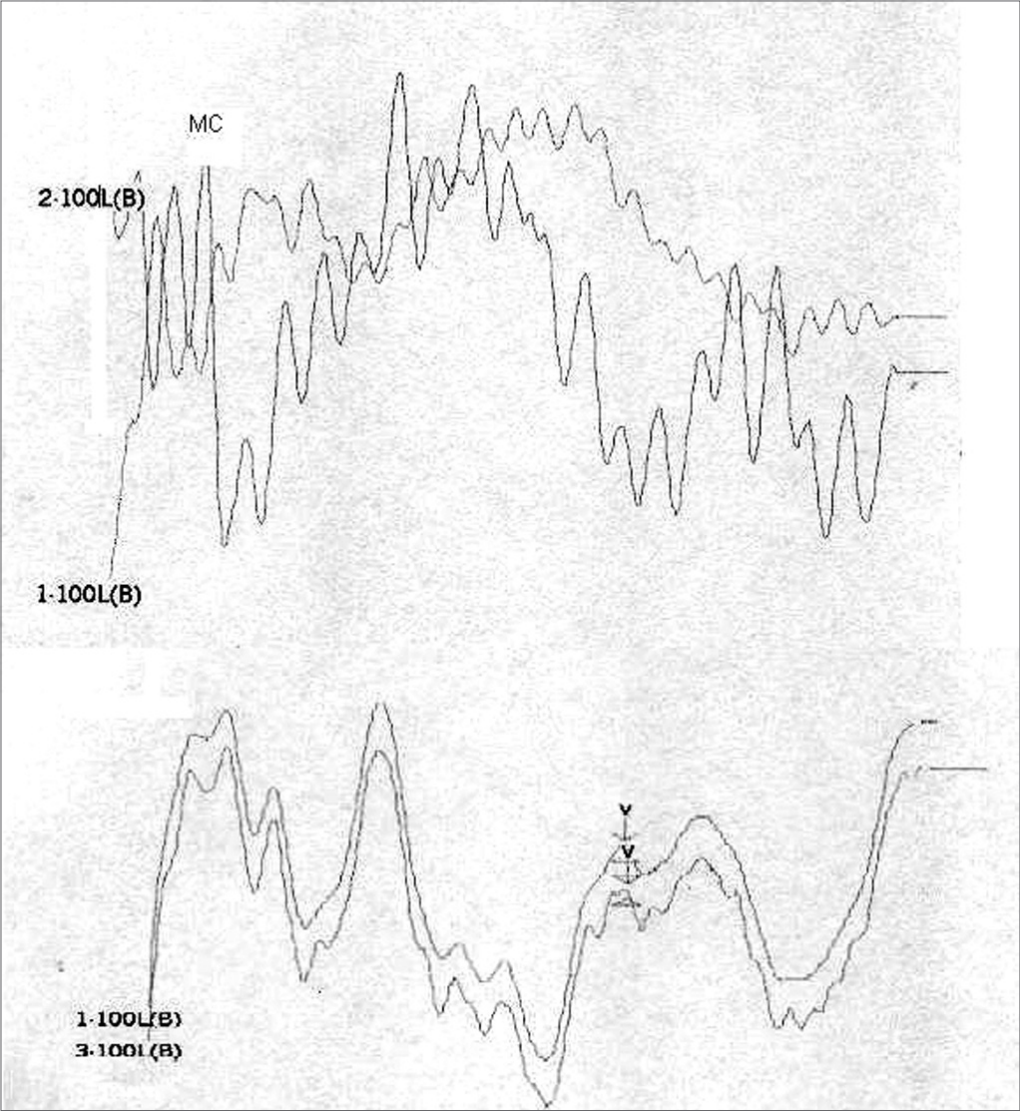
Following stimulus polarity inversion, cochlear microphonism (CM) appears; with a reduced stimulus frequency, wave V appears.

**Case 6, right ear:**
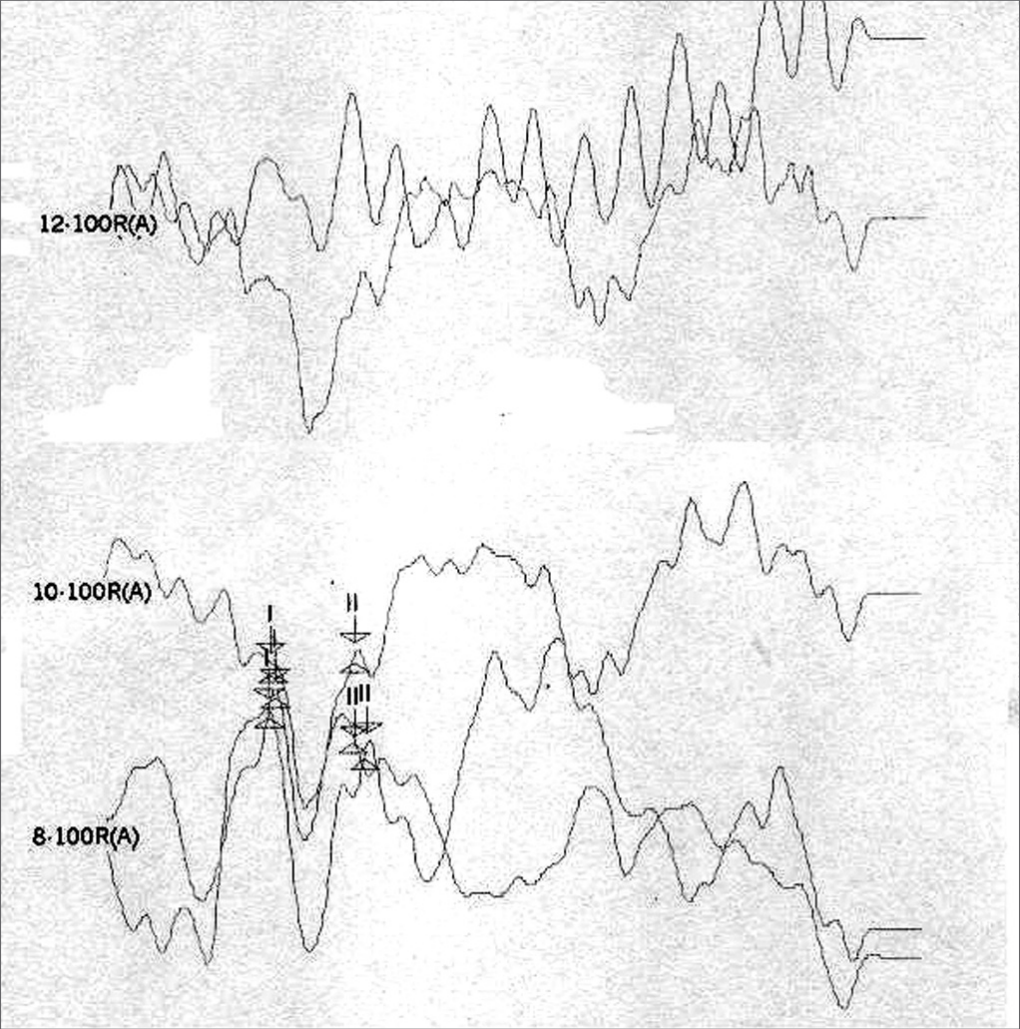
Following stimulus polarity inversion there is no cochlear microphonism (CM); with a reduced stimulus frequency, wave I and II appear.

**Case 6, left ear:**
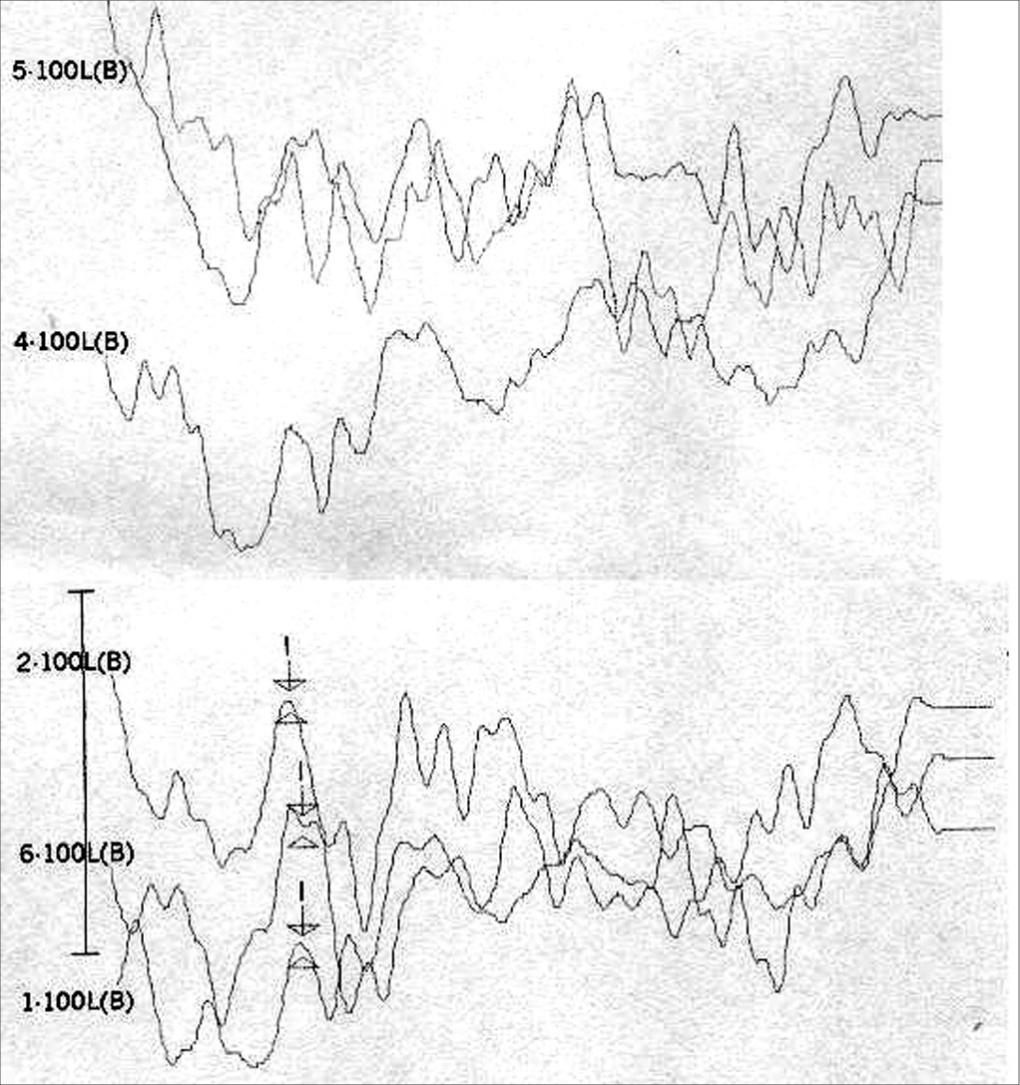
Following stimulus polarity inversion there is no cochlear microphonism (CM); with a reduced stimulus frequency, wave I appear.

**Case 7, left and right ears:**
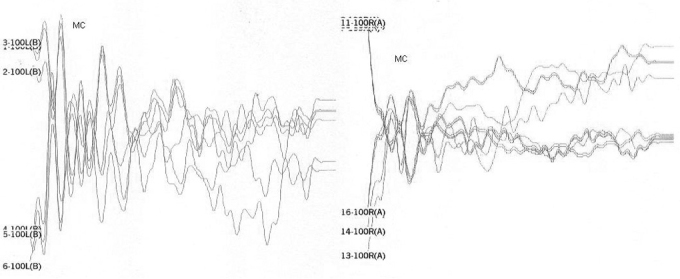
Following stimulus polarity inversion, cochlear microphonism (CM) appears.

## DISCUSSION

There have been many discussions on the early diagnosis and treatment of patients with AN since the description of the first cases. Classically the presence of evoked otoacoustic emission and an absent or significantly altered ABR suggest the diagnosis. However, external ciliated cells may be compromised - leading to OEA loss - in 30% of AN4 individuals, mostly children. There is no consensus about the reasons for this situation, or whether this event is the cause or consequence of neural disorder.^4^,^7^ On the other hand, it is important to differentiate a purely cochlear hearing loss from neural loss with cochlear involvement when faced with a child presenting no clinical response to auditory stimuli, no OEA or ABR, and the need for prompt and precise treatment.

Altered auditory stimuli modify auditory potentials and may offer support in these situations. When there are changes in neural conduction and synchronism, such as in AN, faster stimulus presentation frequencies reduce wave reproduction and clarity. Slower presentation frequencies increase wave amplitude and significantly improve wave patterns.^1^

Stimulus polarity modifications allow visualization of cochlear microphonism, which usually is increased in AN, possibly due to external ciliated cell dysfunction.4 The presence of cochlear microphonism has supported our diagnosis of AN in cases where OEA are absent.

OF the 59 patients in our study, 8 presented altered ABR recordings, showing what we consider a standard response of stimulus polarity and frequency changes as a response to clicks (rarefaction, 27.7 stimuli/sec). Three of these patients had a clear diagnosis of AN (patients 3, 7 and 8), having OAEDP and TOAE, absent ABR, and widened cochlear microphonism. A reduction of stimulus frequency did not improve recordings in these patients, which did not change the diagnosis.

On the other hand, patients 1, 2, 4, and 5 had no OAEDP or TOAE, and presented no electrophysiological response to the standard click, which suggests deep cochlear loss. However, polarity inversion yielded a mirror image typical of cochlear microphonism. the appearance of wave V following a stimulus frequency reduction in patients 2 and 4 reinforced the diagnosis of altered electrical conduction in distal regions of the auditory pathway. Our hypothesis is that these were AN cases that had lost OEA with the progression of the disease. This diagnosis would not have been possible without this protocol. In our view, this is the group of patients that stands to benefit most from the protocol suggested in our study.

Case 6 had no OAEDP, TOAE, electrical response with the standard click, or cochlear microphonism, which suggests deep cochlear loss. However, the appearance of wave I and II following a 3.3 stimulus/sec stimulus frequency suggested alterations of electric conduction in proximal portions of the brain stem, with possible simultaneous cochlear involvement.

The importance of specific topographic diagnosis is its effect on the treatment strategy. Auditory abilities based on electroacoustic devices, the benefits of sound amplification devices and cochlear implants, and the type of language therapy, may all be conducted differently for those cases of cochlear hearing loss compared to those that have neural or central hearing loss.

Although we believe that a topographic diagnosis results from the sum of clinical, psychoacoustic, acoustic, and electrophysiological and image findings, 8 (13.56%) patients benefited from the proposed ABR protocol.

## CONCLUSION

Polarity and frequency changes when presenting clicks during ABR add support to the topographic diagnosis of hearing loss, and should be done in all patients with this condition.
